# Integrated ACMG-approved genes and ICD codes for the translational research and precision medicine

**DOI:** 10.1093/database/baad033

**Published:** 2023-05-17

**Authors:** Raghunandan Wable, Achuth Suresh Nair, Anirudh Pappu, Widnie Pierre-Louis, Habiba Abdelhalim, Khushbu Patel, Dinesh Mendhe, Shreyas Bolla, Sahil Mittal, Zeeshan Ahmed

**Affiliations:** Rutgers Institute for Health, Health Care Policy and Aging Research, Rutgers University, 112 Paterson St, New Brunswick, NJ 08901, USA; Rutgers Institute for Health, Health Care Policy and Aging Research, Rutgers University, 112 Paterson St, New Brunswick, NJ 08901, USA; Rutgers Institute for Health, Health Care Policy and Aging Research, Rutgers University, 112 Paterson St, New Brunswick, NJ 08901, USA; Rutgers Institute for Health, Health Care Policy and Aging Research, Rutgers University, 112 Paterson St, New Brunswick, NJ 08901, USA; Rutgers Institute for Health, Health Care Policy and Aging Research, Rutgers University, 112 Paterson St, New Brunswick, NJ 08901, USA; Rutgers Institute for Health, Health Care Policy and Aging Research, Rutgers University, 112 Paterson St, New Brunswick, NJ 08901, USA; Rutgers Institute for Health, Health Care Policy and Aging Research, Rutgers University, 112 Paterson St, New Brunswick, NJ 08901, USA; Rutgers Institute for Health, Health Care Policy and Aging Research, Rutgers University, 112 Paterson St, New Brunswick, NJ 08901, USA; Rutgers Institute for Health, Health Care Policy and Aging Research, Rutgers University, 112 Paterson St, New Brunswick, NJ 08901, USA; Rutgers Institute for Health, Health Care Policy and Aging Research, Rutgers University, 112 Paterson St, New Brunswick, NJ 08901, USA; Department of Medicine, Robert Wood Johnson Medical School, Rutgers Biomedical and Health Sciences, 125 Paterson St, New Brunswick, NJ 08901, USA

## Abstract

A timely understanding of the biological secrets of complex diseases will ultimately benefit millions of individuals by reducing the high risks for mortality and improving the quality of life with personalized diagnoses and treatments. Due to the advancements in sequencing technologies and reduced cost, genomics data are developing at an unmatched pace and levels to foster translational research and precision medicine. Over 10 million genomics datasets have been produced and publicly shared in 2022. Diverse and high-volume genomics and clinical data have the potential to broaden the scope of biological discoveries and insights by extracting, analyzing and interpreting the hidden information. However, the current and still unresolved challenges include the integration of genomic profiles of the patients with their medical records. The definition of disease in genomics medicine is simplified, whereas in the clinical world, diseases are classified, identified and adopted with their International Classification of Diseases (ICD) codes, which are maintained by the World Health Organization. Several biological databases have been produced, which include information about human genes and related diseases. However, still, there is no database that exists, which can precisely link clinical codes with relevant genes and variants to support genomic and clinical data integration for clinical and translational medicine. In this project, we focused on the development of an annotated gene–disease–code database, which is accessible through an online, cross-platform and user-friendly application, i.e. PROMIS-APP-SUITE-Gene-Disease-Code. However, our scope is limited to the integration of ICD-9 and ICD-10 codes with the list of genes approved by the American College of Medical Genetics and Genomics. The results include over 17 000 diseases and 4000 ICD codes, and over 11 000 gene–disease–code combinations.

**Database URL** https://promis.rutgers.edu/pas/

## Introduction

Symptom-driven medicine has become the domain of medical research in the past decade (1, 2). However, some challenges arise when focusing on the symptoms rather than the disease. Patients with life-threatening diseases might not feel pain and seek professional help. Thus, personalized treatment to help manage and identify those patients using precision medicine is needed to effectively diagnose and provide the most optimal actions needed for such patients (3–5). Precision medicine is a multi-disciplinary field that utilizes the clinical and multiomics data of an individual to create patient-specific treatment plans and diagnoses (4, 7, 8). Clinical data are most familiar to clinicians and patients as a medium that communicates personal and health information between the provider and the patient. Genomic information is stored within various databases that include but are not limited to ClinVar, CNVD, Cochrane Library, Disease Ontology and Disease Enhancer, which allow for gene annotation (4). However, there is a lack of standardized, comprehensive databases that consolidate the known gene–disease relationships. Furthermore, there is no known database that connects International Classification of Diseases (ICD), mediated by the World Health Organization (WHO), with the list of 73 genes compiled by the American College of Medical Genetics and Genomics (ACMG), whose mutations are known to be causative of disorders and diseases (9).

The evolution from the first use of the word ‘gene’ to our current understanding has launched a new scientific age. On an introductory level, the chemical structure of the genome is in the form of deoxyribose nucleic acid (DNA), which is composed of a double helix with pairs of nucleotides connected by hydrogen bonds (1, 10, 11). These alternating patterns of nucleotides (adenine, cytosine, guanine and thymine) encode the instructions for all the proteins in our body, yet only a fraction of the entire genome contains protein-coding sequences (6, 12). The goal of genomic medicine is to isolate and examine the mutations in these sequences that lead to diseases (6, 13, 14). This objective is observable in the link between sickle cell anemia and the mutation in the protein encoding haemoglobin once the genome is sequenced (1). The sequencing and understanding of these mutations have been made possible by Next-Generation Sequencing (NGS) (15). Currently, Illumina sequencing is the most popular sequencing technology due to its accuracy, cost and speed (16). Illumina sequencing belongs to a family of NGS technology that produces short reads (50–300 base pairs), with the most notable other technology in this category being Ion Torrent sequencing (17). After the sequencing data are collected, they are displayed and shared as a FASTQ file. Each sequence stored in the FASTQ file has four corresponding lines of text. These lines contain information such as the sequence identifier, nucleotide sequence, a ‘+’ sign to indicate the end of the sequence and a line of quality values reported in the American Standard Code for Information Interchange characters (6, 18). Using gene information in a FASTQ file, algorithms map the reads to the reference genome and store it in a Sequence Alignment Map file or its Binary Equivalent Map file (19). From the Sequence Alignment Map file, variant call format files are created, which store information regarding variations, insertions and deletions (6). Whole Genome Sequencing (WGS) and Whole Exome Sequencing (WES) are two types of NGS that are more accurate methods of DNA sequencing and are used to find variants in a DNA sequence (20). While WGS sequences the whole genome, WES sequences only the protein-coding regions (21).

Recent developments in sequencing technologies have greatly aided in long-read sequencing and integration of genomic data. However, challenges arise when integrating heterogenous data such as clinical and genomic data. Electronic health records (EHRs) contain a large volume of data that cannot be processed at a fast and efficient rate on local servers. Thus, it is vital to use high-performance computing to process these data (22). We recently created a Java-based Whole Genome/Exome Sequence Data Processing Pipeline (JWES), a free, open-source pipeline that processes and analyzes WES/WGS data (23). Due to the personal nature of the data included in EHRs, it is imperative that safeguards are placed to protect the confidentiality of such data (24). In the genetics field, ACMG is a medical organization that is responsible for guidelines internationally accepted for variant interpretation along with improving health through genomics and medical genetics (9). ACMG is responsible for publishing and providing recommendations for clinical exome and genome sequencing that provides a universally accepted platform for scientists to work and discover any new incidental findings (9). Presently, 73 genes have been proposed by ACMG, which are known to be of importance to disorders and can be clinically acted on by an accepted way of intervention (25). These genes provide significant medical value as they allow for improved clinical treatment (9, 25).

The duality of information stored by genomic and clinical data in a single network would form a comprehensive patient profile that creates the possibility for individualized health care. However, there is no system that integrates the two data types and standardizes the data according to international academic standards (1, 23, 26). This shortcoming allows symptom-based treatments to be normalized as the default approach to patient care, and to challenge the standard model, a solid connection must be made between clinical and genomic data (23, 26). Even with the latest sequencing technologies, the format and robustness of raw genomics data is not well suited for current EHR systems (27). Raw genomic files must undergo various processing procedures before being able to be visualized and used by non-bioinformaticians (23). Combined with the intense computing environment needs for maintaining an EHR system (22), there is also an infrastructural component to consider. However, recent developments in the field highlight some promising outcomes in the creation of a unified genomic-EHR system. PROMIS-APP-SUITE (PAS)-Gen mobile application is a publicly available iOS app that leverages a database of over 59 000 coding and non-coding genes along with 90 000 gene–disease associations (20). It was created with the intention of assisting academic researchers and medical professionals in understanding the dynamic between disease and genes (20).

The organization of health care information is largely based on a label-based system. On a global scale, the WHO created standardized ICD codes, while the Food and Drug Administration maintains the National Drug Code (28). National Drug Code serves as an identifier for prescription and over-the-counter drugs as well as insulin. This database contains information pertinent to the commercial sale of drugs, including manufacturer and packing details (29). In this project, we have designed and implemented a relational database and interactive online web application that connects genomic and clinical data, allowing a user to discover the relationship between genes and diseases along with their respective ICD codes. We hypothesize that our web application can assist healthcare providers and clinicians in creating a more personalized treatment approach by observing gene–disease–ICD. However, the scope of research is limited only to the ACMG-approved genes.

## Materials and methods

Our methodology was divided into three main sections. First, we focused on curation and integration of the genomic and clinical data. Then, we focused on designing and modelling of a new relational database to facilitate data manipulation. The last step highlights the implementation of our efficient and user-friendly online web application PAS-Gene-Disease-Code (GDC) to facilitate an integrated search of clinical and genomic data all in one place.

### Gene–disease–code data curation and integration

**Figure 1.**
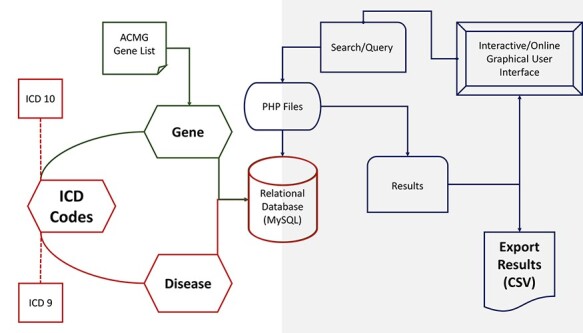
PAS-GDC components’ design, development and data flow. PAS-GDC is an online application developed using MySQL database, PHP scripting language and UNIX-based web and database servers.

PAS-GDC website uses 73 approved genes from ACMG as well as the ICD codes to curate the data that power the search engine (Table 1). ICD-9 and ICD-10 codes were utilized in the creation of our relational database. While the ICD-9 codes were proposed by the WHO to provide a unified system to present mortality statistics, the ICD-10 codes were implemented for inpatient procedures in hospitals (28). Additionally, the structures of ICD-9 and ICD-10 codes are vastly different, where ICD-9 codes are numeric, and ICD-10 are alphanumeric. The PAS-GDC website currently holds 2101 ICD-9 codes and 2589 ICD-10 codes that were manually curated for search functionalities. Additionally, there are 7918 and 11 799 gene–disease combinations for ICD-9 and ICD-10 codes, respectively (Table 2). Two Excel sheets containing up-to-date information regarding each of the 73 actionable genes, their relevant diseases and relevant ICD-9 and ICD-10 codes were curated. For easy translation from the Excel sheet to Structured Query Language (SQL) relation, a Python extraction, transfer and loading script was written. Running this script provides the user with a text file containing the genes, diseases and ICD codes, which can be copied and pasted into SQL to create two relations containing all information from both Excel sheets (Figure 1). The current version of the PAS-GDC includes the complete release of ICD-10 and ICD-9, provided by the WHO.

**Table 1.** List of American College of Medical Genetics and Genomics (ACMG) genes

Number	Genes	Name of the disease
1	*BRCA1*	Hereditary breast and ovarian cancers
2	*BRCA2*	
3	*PALB2*	
4	*TP53*	Li-Fraumeni syndrome
5	*STK11*	Peutz-Jeghers syndrome
6	*MLH1*	Lynch syndrome
7	*MSH2*	
8	*MSH6*	
9	*PMS2*	
10	*APC*	Familial adenomatous polyposis
11	*MUTYH*	MYH-associated polyposis, adenomas, multiple colorectal, FAP type 2, colorectal adenomatous polyposis, autosomal recessive, with pilomatricomas
12	*BMPRA1*	Juvenile polyposis
13	*SMAD4*	
14	*VHL*	Von Hippel–Lindau syndrome
15	*MEN1*	Multiple endocrine neoplasia type 1
16	*RET*	Multiple endocrine neoplasia type 2 and familial medullary thyroid cancer
17	*PTEN*	*PTEN* hamartoma tumor syndrome
18	*RB1*	Retinoblastoma
19	*SDHD*	Hereditary paraganglioma-pheochromocytoma syndrome
20	*SDHAF2*	
21	*SDHC*	
22	*SDHB*	
23	*MAX*	
24	*TMEM127*	
25	*TSC1*	Tuberous sclerosis complex
26	*TSC2*	
27	*WT1*	*WT1*-related Wilms tumor
28	*NF2*	Neurofibromatosis type 2
29	*COL3A1*	Vascular-type Ehlers-Danlos syndrome
30	*FBN1*	Marfan syndrome, Loeys-Dietz syndromes and familial thoracic aortic aneurysms and dissections
31	*TGFBR1*	
32	*TGFBR2*	
33	*SMAD3*	
34	*ACTA2*	
35	*MYH11*	
36	*MYBPC3*	Hypertrophic cardiomyopathy, dilated cardiomyopathy
37	*MYH7*	
38	*TNNT2*	
39	*TNNI3*	
40	*TPM1*	
41	*MYL3*	
42	*ACTC1*	
43	*PRKAG2*	
44	*GLA*	
45	*MYL2*	
46	*LMNA*	
47	*FLNC*	
48	*TTN*	
49	*RYR2*	Catecholaminergic polymorphic ventricular tachycardia
50	*CASQ2*	
51	*TRDN*	
52	*PKP2*	Arrhythmogenic right ventricular cardiomyopathy
53	*DSP*	
54	*DSC2*	
55	*TMEM43*	
56	*DSG2*	
57	*KCNQ1*	Romano-Ward long-QT syndrome types 1, 2 and 3 and Brugada syndrome
58	*KCNH2*	
59	*SCN5A*	
60	*LDLR*	Familial hypercholesterolemia
61	*APOB*	
62	*PCSK9*	
63	*ATP7B*	Wilson disease
64	*OTC*	Ornithine transcarbamylase deficiency
65	*BTD*	Biotinidase deficiency
66	*GAA*	Pompe disease
67	*RYR1*	Malignant hyperthermia susceptibility
68	*CACNA1S*	
59	*HFE*	Hereditary haemochromatosis
70	*ACVRL1*	Hereditary haemorrhagic telangiectasia
71	*ENG*	
72	*HNF1A*	Maturity-onset diabetes of the young
73	*RPE65*	*RPE65*-related retinopathy

A list of 73 ACMG-approved genes for which specific mutations are known to be causative of disorders with defined phenotypes that are clinically actionable by an accepted intervention is included. The disease phenotype associated with each gene is also included.

**Table 2.** PAS-GDC database description and statistics

Categories	Count
Genes	73
Diseases	1788
ICD-9	2101
ICD-10	2589
Gene–disease combination (ICD-9)	7918
Gene–disease combination (ICD-10)	11 799

PAS-GDC database includes genes, diseases and ICD-9 and ICD-10 codes, as well as the relevant gen–disease combinations for each ICD code.

### Relational database modelling

**Figure 2.**
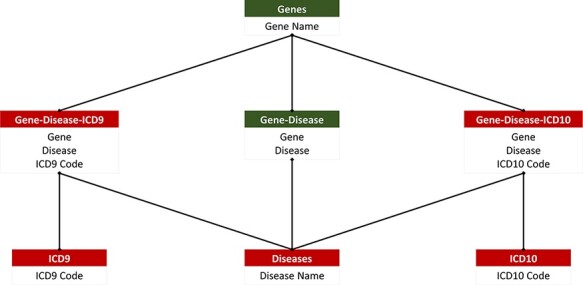
PAS-GDC relational database. PAS-GDC database includes six relations, genes, diseases, ICD9, ICD10, gene–disease, gene–disease–ICD-9 and gene–disease–ICD-10.

The main objective of the database was to make the compiled information easily searchable and parsed so that all searches from the website would be up to date. Additionally, the database design is needed to support easy integration of future ICD codes to ensure that up-to-date information is reflected in our website. To meet these requirements, the database was created in MySQL (open-source relational database management system) Workbench and consisted of seven relations. The seven relations included ACMG’s 73 actionable genes, diseases, ICD-9 codes, ICD-10 codes, gene–disease pairings, gene–disease–ICD-9 pairings and gene–disease–ICD-10 pairings. The gene–disease–ICD-9 and gene–disease–ICD-10 relations are created from the relations that manage the genes, diseases and their respective ICD codes (Figure 2). This ensures that there are no duplicate values. This database is unique and accessible through our freely available, open-source web application.

### Web development and search

**Figure 3.**
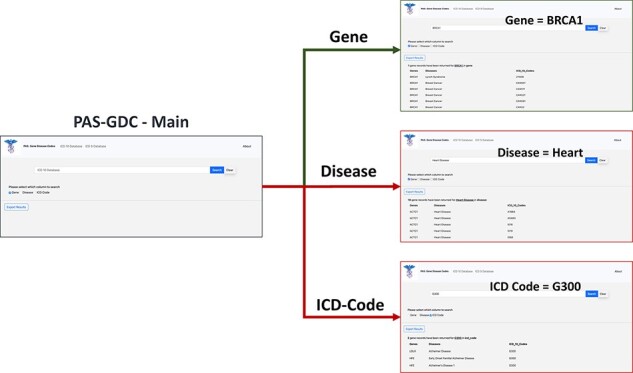
PAS-GDC graphical user interfaces workflow. PAS-GDC GUI includes Main, Gene, Disease and ICD Code (9 and 10) interfaces.

PAS-GDC is a web application that has been developed using Hypertext Markup Language (HTML) and JavaScript with its jQuery packages. Additionally, we have used Cascading Style Sheets with a Bootstrap framework on HTML to enhance the presentation and provide a user-friendly interface to our users. The database is connected to our web application using server-side PHP (general-purpose scripting language for web application development) language and its ‘mysqli’ package. Visual Studio Code was the primary Integrated Development Environment used in the creation of the source code as well as testing. The testing of the website involved using Red Hat localhost servers. During development, testers used macOS, iOS, Windows and Android operating systems along with a variety of different browsers that include but are not limited to Google Chrome, Safari and Firefox to ensure that the website performs typically and is configured correctly regardless of the environment. SSL certificates were utilized in the PAS-GDC website, and the communication between the browser and server was encrypted. The search allows the user to perform searches based on the ICD-9 codes, ICD-10 codes, gene or disease category (Figure 3). The ICD-9 and ICD-10 searches allow for their independent gene and disease search allowing the user to retrieve the respective gene–disease pairing based on the desired ICD selection. Additionally, the website allows for a simple and easy export feature that allows the users to store and share their desired results as a comma-separated values (CSV) file. The user interface of PAS-GDC is explained in the supplementary material attached.

## Results

The gene–disease–ICD code database is a flexible and dynamic database. The database design in SQL allows for more genes and ICD codes to be integrated as they are made available and updated automatically on the PAS-GDC website. PAS-GDC is a simple-to-use, robust search engine that utilizes minimalistic features and an internet connection to retrieve results. The graphical user interface includes a search capability of three features, namely (1) ICD Codes, (2) Genes and (3) Diseases as a simple check box that gives the users the capability to choose the feature they desire. Additionally, PAS-GDC provides the users the option to search their results against ICD-9 codes and ICD-10 codes.

### Case study: gene

**Figure 4.**
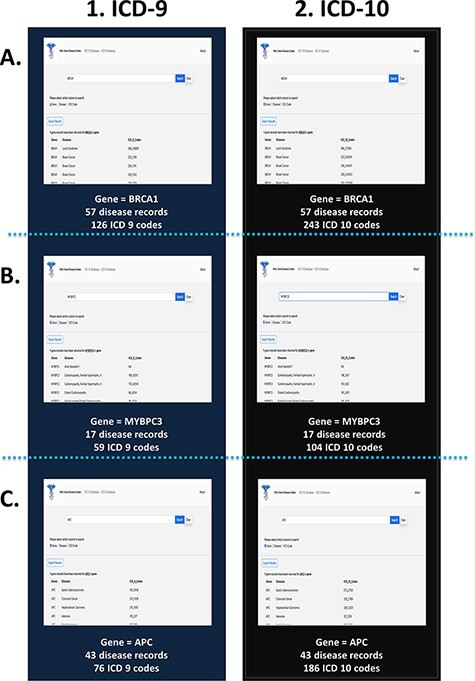
PAS-GDC use case—gene. This figure presents three different case studies exploring the ‘gene’ search feature: *BRCA1* (A), *MYBPC3* (B) and *APC* (C).

To test the effectiveness and functionality of the PAS-GDC web application, we created three different case studies exploring the ‘gene’ search feature (Figure 4). The genes that were included in this case study were *BRCA1, MYBPC3* and *APC*. The results were exported and collected in a tabular format with three columns: genes, diseases and ICD codes. The *BRCA1* gene codes for proteins that are vital to a multitude of cellular processes (30). Mutations in this gene can lead to a predisposition to breast and ovarian cancers (30–32). The search results for the *BRCA1* gene present 57 distinct diseases that are directly linked to this gene. These diseases include but are not limited to breast, ovarian and pancreatic cancers as well as fallopian tube carcinoma. Additionally, the search uncovered a total of 126 ICD-9 (Figure 4A(1)) and 243 ICD-10 codes associated with *BRCA1* (Figure 4A(2)). ICD-9 codes starting with 17 seemed to repeat for the *BRCA1* gene as this category denotes breast cancer. Similarly, ICD-10 codes starting with C4 and C5 were the most common in the search. The search criteria were repeated for the gene *MYBPC3*. Mutations in this gene are usually linked to cardiovascular diseases such as cardiomyopathy and atrial fibrillation (33). Seventeen other diseases were also found to be linked to this gene through our web application. These diseases included but were not limited to diastolic heart failure, cardiac arrest and heart disease. Currently, there are 59 ICD-9 (Figure 4B(1)) and 104 ICD-10 codes linked to *MYBPC3* (Figure 4B(2)). One of the most common diagnoses linked to this gene was cardiovascular disease (heart disease), the leading cause of death in the USA (34, 35). Thirty-three of the fifty-nine ICD-9 codes and fifty-eight of the 104 ICD-10 codes are linked to heart disease showing their prevalence and impact on patients with a genetic mutation in the *MYBPC3* gene. The third case study focused on the *APC* gene, which is known to lead to a predisposition to colorectal and lung cancers (36, 37). Based on the results from the PAS-GDC web application, it was observed that there are 43 diseases that are associated with this gene. Some of the diseases highlighted in the results included but were not limited to lung cancer , thyroid cancer (38) and breast cancer (39). Seventy-six ICD-9 (Figure 4C(1)) and 186 ICD-10 codes were retrieved for the *APC* gene (Figure 4C(2)). Notably, the most common diagnoses linked to this gene included breast and lung cancers. While APC had been linked to lung cancer in previous studies, the relation with breast cancer has not yet been established. Sixteen out of the seventy-six ICD-9 and eighty out of the 186 ICD-10 codes for the *APC* gene were associated with breast cancer.

### Case study: disease

**Figure 5.**
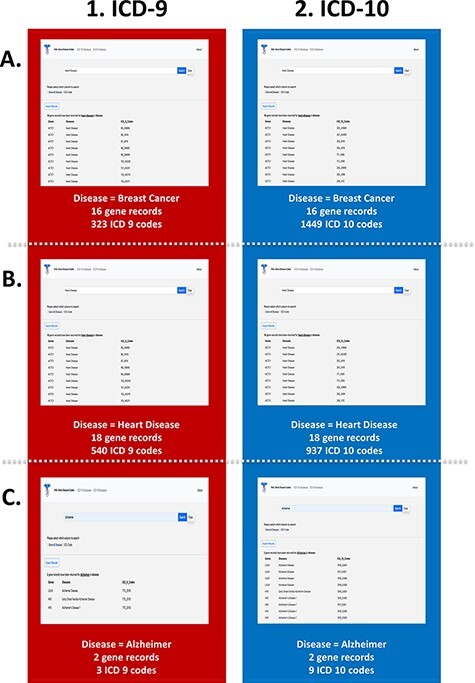
PAS-GDC use case—disease. This figure presents three different case studies exploring the ‘disease’ search feature: breast cancer (A), heart disease (B), and Alzheimer’s disease (C).

The link between common disease nomenclature and international classification was exemplified through three case studies of breast cancer, heart disease and Alzheimer’s disease (Figure 5). The disease case studies were chosen because of their prevalence in the general population. Breast cancer is one of the leading causes of death for women worldwide and has an incidence of 1 in 10 cancer diagnoses each year (39, 40). Our web application highlights the impact of this disease by returning 323 ICD-9 (Figure 5A(1)) and 1449 ICD-10 codes (Figure 5A(2)). Additionally, a total of 16 gene records were retrieved for breast cancer, which includes but is not limited to *BRCA1, RB1, APC* and *PTEN*. Like cancer, the term ‘heart disease’ encompasses several different subtypes, and one of the most common forms, congenital heart disease, continues to be a growing burden on health care systems (41). A search of heart disease retrieved 540 ICD-9 (Figure 5B(1)) and 937 ICD-10 codes (Figure 5B(2)). A total of 18 gene records were retrieved for heart disease, the most common gene being *ACTC1*, which has been documented to cause cardiomyopathies (42). Our final case is Alzheimer’s disease that is characteristically prevalent in older adults, and current research implicates a complex relationship between genetic and environmental factors (43). The search yielded three ICD-9 (Figure 5C(1)) and nine ICD-10 codes (Figure 5C(2)). Additionally, two gene records, *LDLR* and *HFE*, were associated with this disease. While these genes have been studied in other forms of neurological diseases, their effects on and interactions with neurodegenerative diseases are not as widely studied. Since ICD-10 diagnostic code set allows for greater specificity in the disease aetiology, anatomic site and severity (44), there are a greater number of codes available, as seen in all three case studies.

### Case study: ICD code

**Figure 6.**
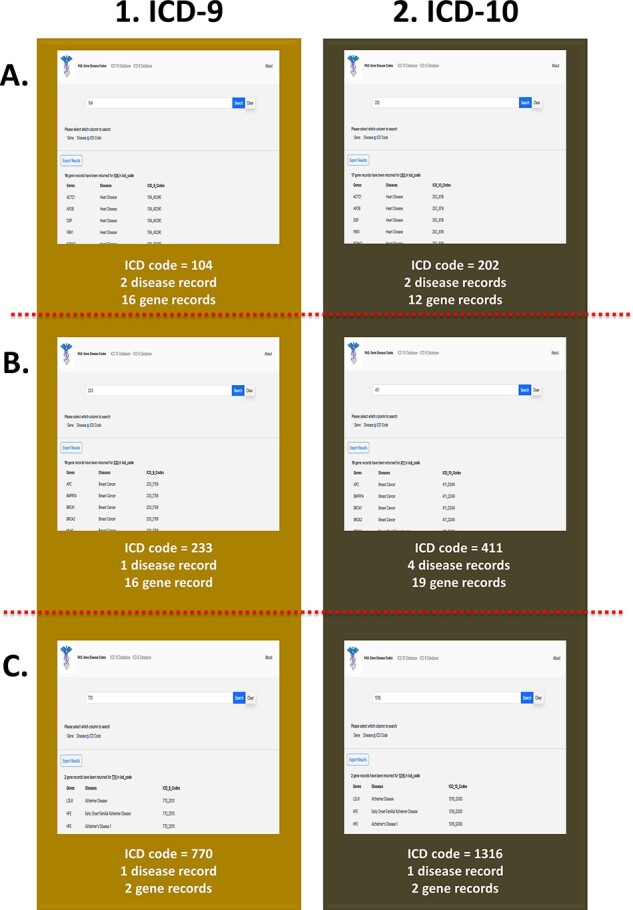
PAS-GDC use case—ICD code. This figure presents three different case studies exploring ICD-9 and ICD-10 codes. (A) The results for ICD-9 and ICD-10 codes starting with ‘104’ and ‘202’, respectively. (B) The results for ICD-9 and ICD-10 codes starting with ‘233’ and ‘411’, respectively. (C) The results for ICD-9 and ICD-10 codes starting with ‘770’ and ‘1316’, respectively.

The third search feature utilized by our web application is based on the ICD codes. The three ICD-9 codes that were included in this case study were 104, 233 and 770. A search on our web application for the ICD-9 code 104 returns 16 unique genes, which include but are not limited to *ACTC1, APOB, PKP2* and *MYH7*, as well as to two common diseases, heart disease and bone fracture (Figure 6A(1)). Notably, the *ACTC1* gene is also associated with other forms of cardiovascular disease as stated previously. The ICD-9 code, 233, yields one distinct disease, breast cancer, which is the most common type of cancer (Figure 6B(1)). Additionally, the search returns 16 unique genes, which include but are not limited to *APC, BRCA1, MLH1, PTEN* and *RB1*. A search in our third case study for the ICD-9 code, 770, shows that this code is associated with Alzheimer’s disease as well as with two distinct genes, *LDLR* and *HFE* (Figure 6C(1)), which have been observed to be linked to Alzheimer’s disease based on our previous queries. We also utilized our ICD-10 database for three distinct case studies involving the codes 202, 411 and 1316. A search for the ICD-10 code, 202, returns 12 unique genes and 2 diseases, heart disease and ptosis (Figure 6A(2)). The code, 411, is associated mainly with breast cancer as well as other phenotypic variations of this disease such as breast giant fibroadenoma and breast benign neoplasm. Additionally, the search yields 19 distinct genes, which are linked to this unique ICD code (Figure 6B(2)). The code 1316 is linked to one distinct disease, Alzheimer’s, and two genes, *LDLR* and *HFE* (Figure 6C(2)).

The connection to our in-house database does not require the user to install any tools or external modifications. When users search for their desired keywords, it triggers the database and cross-references for the exact or similar keywords. Once the database retrieves the results, it is presented to the user in a table format displaying the gene, disease and ICD code as separate columns. Additionally, our web application allows users to save their desired results as a text (CSV) file. The intelligent search feature of the PAS-GDC removes the need to cross-verify genes or diseases on other web applications or databases by integrating and providing an all-in-one (gene, ICD and disease) search capability to the users. Updates to the database might include but are not limited to new additions to the ACMG-approved genes, new associations between genes and diseases and the addition of another version of the ICD code.

## Discussion

Recent developments in sequencing technologies and analysis of gene expression and variant data have helped advance the field of precision medicine (37). Genomic and transcriptomic analyses have the potential to be a driver for clinically reliable predictions of complex diseases and disorders. In a clinical research setting, the exploratory and dynamic nature of precision medicine yields promising results in discovering new gene–disease relationships, variants and diverse genotyping (3). NGS has aided in the implementation of personalized treatments for patients with cardiovascular disease and neurodegenerative conditions (3). Some of the applications of NGS include but are not limited to genomic data models to support clinical decision-making, identification of robust epigenetic biomarkers as well as clinical translation (3). Additionally, the latest research indicates that there is merit in integrating untargeted metabolomic profiling with genomic analysis for individuals at the ends of phenotypic expression (26). This approach demonstrates that integrated genomics helps narrow the gap between treatment and disease by leveraging streamlined analysis of a patient’s genome, thus saving critical diagnosis time and money for the patient and institution of care (45). However, there are still many constraints when trying to integrate genomic and clinical data. These constraints include but are not limited to lack of standardization when linking genes to their disease phenotypes (20), difficulty in integrating huge amounts of genetic and clinical data (46) and absence of a platform that contains up-to-date genome and clinical data (20, 47, 48). To address these limitations, we have created PAS-GDC, a web application, that is easy to navigate and freely available on many platforms. This graphical user interface was designed so that it can be used by non-computational users, such as physicians and geneticists, allowing for the integration of precision medicine in the clinical field. Beyond a computational lens, clinicians and patients can interpret clinical and genomic data by learning the implications of one or more mutations in the genome and present actionable steps in a more effective, personalized treatment plan. Additionally, researchers in various fields could use our web application to support their work, especially those seeking connections between genomics and phenotypical manifestation.

One of the immediate implications of our web application is to support the downstream bioinformatic analysis involving gene–disease relationships. The produced outcome of the PAS-GDC is based on the integrated information including authentic and ACMG-approved genes, WHO-provided ICD codes and associated diagnoses. To the best of our knowledge, so far, there is no comprehensive, dedicated, user-friendly, manually curated and detailed application exists, which is mainly proposed, designed and developed to share such important information for supporting the objectives of translational research and precision medicine (49). One of the most important benefits of the outcome of PAS-GDC is to help in filling the gaps between basic sciences and clinical research. Most of the time, the outcomes of the research based on the high-throughput genomics data analysis cannot be linked to the EHR of the patients, precisely. The reason for this is that in the clinical world, one disease can be represented with several phenotypes and classified using variable ICD codes available through the different health systems, e.g. EPIC, NextGen and Cerner. To efficiently link the outcomes of genomics research, it is important to first classify authentic disease-causing genes and then link those to the relevant ICD codes. There are many advancements, when it comes to the development of bioinformatic tools and the application of machine learning and Artificial Intelligence approaches for the processing, analysis and integration of clinical data with multi-omics/genomics data (6, 26, 46). Here, the challenges and limitations are when it comes to usability and interpretation, as most of such applications require strong computation background from their users. Furthermore, they might need its users to install, learn and practice programming languages (e.g. R, Python, SAS and MATLAB), relational databases (e.g. MySQL and Microsoft SQL), high-performance and secure computing environments (e.g. cloud and data cluster) and handling of text files of variables sizes and structures. This can increase their time to access and interpret data, cost to afford and reduce the liberty to exercise and experiment with it. However, the availability and application of user-friendly platforms such as PAS-GDC will help addressing such challenges at global levels and can benefit millions of users from diverse backgrounds expertise.

The fundamental aspect of our PAS-GDC database is the 73 ACMG codes as well as the ICD-9 and ICD-10 codes. The backend development was labor-intensive, and we chose actionable genes that have been shown to be causative of disorders as a strategic start. There are seven relationship databases created to compile the information cohesively and serve as a base for future updates, allowing our web application to remain up to date. To optimize our process, we are exploring different methods to address the time-consuming aspect of data curation. Furthermore, we are interested in using Artificial Intelligence and machine learning algorithms for data mining. A solid foundation was created, and the tools to build out the database to a more robust capacity are readily available. We are extending the scope of our project by implementing more disease-causing genes in our database as well as different versions of the ICD code as they are made available. With the copious amounts of data available and the development of systems that can interpret them on a large scale, the focus of treatment can shift from symptom-based to prevention and early intervention in unprecedented ways. A world with precision medicine would challenge the current health care system by centering care on maintaining health instead of addressing the lack thereof.

## Conclusion and future recommendations

PAS-GDC is a cross-platform online application compatible with Microsoft Windows and macOS operating systems. It has been developed using relational database management systems and programming languages including but not limited to HTML/Cascading Style Sheet with Bootstrap Framework, PHP, MySQL and JavaScript. The current version of PAS-GDC is based on the set of 73 genes approved by the ACMG. In the future, we are looking forward to increase the number of genes with the identification and inclusion of more authentic genes and variants, produced as the result of genome-wide association studies (GWAS) (50, 51) and other important analyses. PAS-GDC can execute in smartphones (e.g. iOS and Android-based devices) but using installed web browsers. To increase its visibility, application and benefits at the global level, in the future, we aim to develop the desktop and iOS-based user interfaces of PAS-GDC and integrate them into the PROMIS-APP-SUITE (25) and Visualizing Genes with disease causing Variants (52). PAS is an iOS application designed to simplify navigation across the landscape of gene annotation resources by an efficient mobile record search engine, which is based on standardized genes and related diseases to help explore multi-purpose clinical and genomics concepts in meaningful ways (25).

## Supplementary Material

baad033_Supp

## Data Availability

Data are freely available and can be accessed via a https://promis.rutgers.edu/pas/. All results produced and discussed in this article are incorporated into the article and its online supplementary material.
