# Supplementary material for: Integrated ACMG-approved genes and ICD codes for the translational research and precision medicine
Source: Database (Oxford). 2023 May 17;2023:baad033. doi: 10.1093/database/baad033 (PMC10191139; doi:10.1093/database/baad033)
Supplement: baad033_Supp [file baad033_supp.zip › suppl_data/Supplementary_Material_1_Tutorial_04132023.docx]

**Supplementary Material 1:**

PAS-GDC: Tutorial and Relational Database Modelling

**Manuscript Title:**

Integrated ACMG approved genes and ICD codes for the translational research and precision medicine

**Authors**

Raghunandan Wable^1, †^, Achuth Suresh Nair^1, †^, Anirudh Pappu^1, †^, Widnie Pierre-Louis^1, †^, Habiba Abdelhalim^1, †^, Khushbu Patel^1, †^, Dinesh Mendhe^1^, Shreyas Bolla^1^, Sahil Mittal^1^, and Zeeshan Ahmed^1, 2, 3*^

**Affiliations**

1. Rutgers Institute for Health, Health Care Policy and Aging Research, Rutgers University, 112 Paterson St, New Brunswick, NJ, USA.

2. Department of Medicine, Robert Wood Johnson Medical School, Rutgers Biomedical and Health Sciences, 125 Paterson St, New Brunswick, NJ, USA.

^†^Equally contributing first authors.

***Corresponding author:** Zeeshan Ahmed, Rutgers Institute for Health, Health Care Policy and Aging Research, Rutgers University, 112 Paterson Street, New Brunswick, 08901, NJ, USA. ([zahmed@ifh.rutgers.edu](mailto:zahmed@ifh.rutgers.edu)).

**Table of Contents**

[PAS: Gene Disease Database (GDC) 3](#_Toc120483263)

[Gene Disease Database: Home Page 3](#_Toc120483264)

[Gene Disease Database: Searching 4](#_Toc120483265)

[Searching Using ICD Codes 4](#_Toc120483266)

[**ICD 10** 4](#_Toc120483267)

[**ICD 9** 5](#_Toc120483268)

[Searching Using Gene Checkbox 6](#_Toc120483269)

[Searching Using Disease Checkbox 7](#_Toc120483270)

[Exporting Results 8](#_Toc120483271)

[Gene Disease Database: Graphical User Interface 9](#_Toc120483272)

[Software Development 10](#_Toc120483273)

[Gene Disease Database 10](#_Toc120483274)

[References 11](#_Toc120483275)

# **PAS: Gene Disease Database (GDC)**

Gene Disease Database is a user-friendly webpage developed by Ahmed Lab which allows users to search genes, diseases as well as ICD 9 and ICD 10 codes. The webpage features a search bar with simple checkboxes that will allow the user to retrieve the results they desire. Additionally, the webpage allows the user to export retrieved search results in a CSV file (.csv) for their desired use. The results are provided in an organized manner featuring a gene, disease, and ICD column. The ICD codes can be selected on the webpage according to the user’s wish if they desire ICD 9 or ICD 10 Codes. Gene Disease Database was programmed using PHP, SQL, and HTML. The design of the web page allows anyone to utilize the features without significant technical knowledge, and it can operate on any Internet Browser with a stable network connection. The overall graphical user interface (GUI) consists of the following features (S. Figure 1):

1. Search Bar
2. Text buttons for ICD Codes
3. Check box
4. Results

## **Gene Disease Database: Home Page**


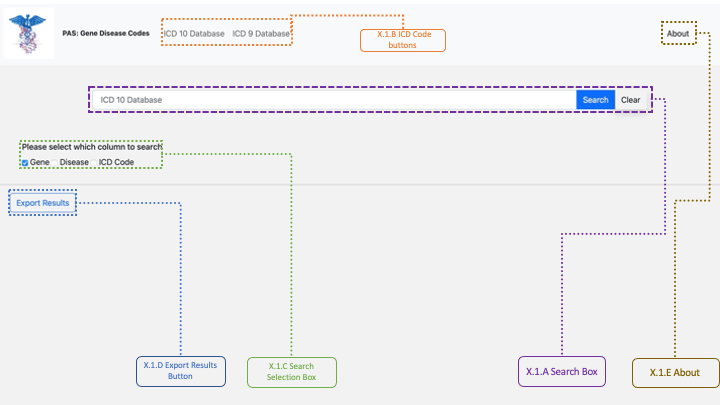


**S.** Figure 1. Gene Disease Database Home page

| **No.** | **Feature** | **Description** |
| --- | --- | --- |
| S.1.A | Search Box | - Text Box for typing Gene/Disease/ICD keywords. - Search button to submit query. - Clear button to clear the text box. |
| S.1.B | ICD Code buttons | - Switch the database between ICD 9 or 10 codes. |
| S.1.C | Search Selection Check Box | - Selects the desired feature to be searched (Gene, Disease, ICD Code). |
| S.1.D | Export Results Button | - Exports Result as CSV file (.csv). |
| S.1.E | About | - Team involved in the development of the project. |

**S.** Table 1. Gene Disease Database home page features

## **Gene Disease Database: Searching**

Gene Disease Database offers an extensive range of search capabilities to tune the results according to the user’s interest and end goal. The user simply needs to input their search keyword (full or partial) and assign the checkboxes and select the text button (ICD 9 or ICD10) according to their interest. The information will then be relayed to our in-house backend connected gene disease database that will retrieve the results requested by the user.

## **Searching Using ICD Codes**

### **ICD 10**


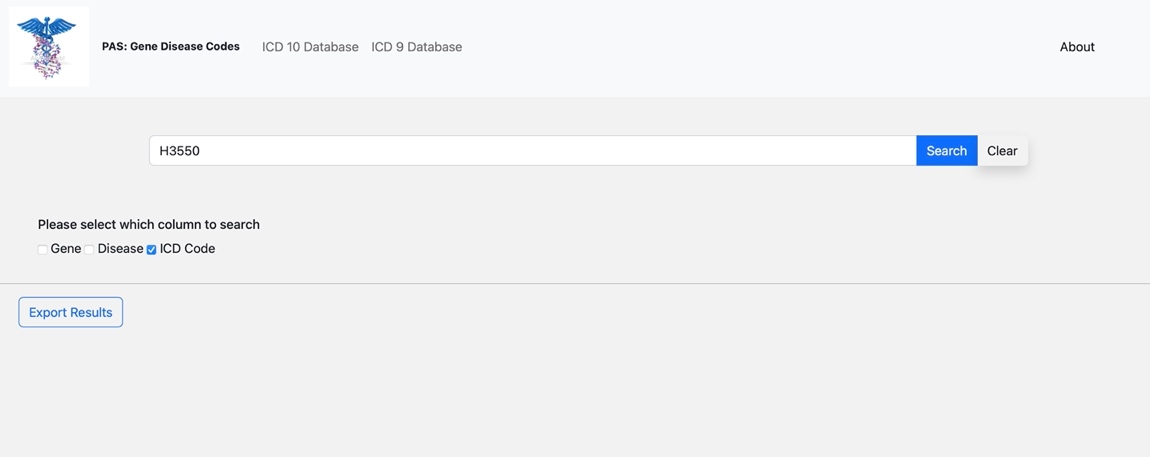


**S.** Figure 2. Searching of ICD 10 Code (H3550)

When the user clicks the ICD 10 text button and the ICD column check box, the webpage will require the input of the ICD 10 code in the search bar by the user. The information will be relayed to our database and provide results based on the options chosen and the ICD code input by the user.


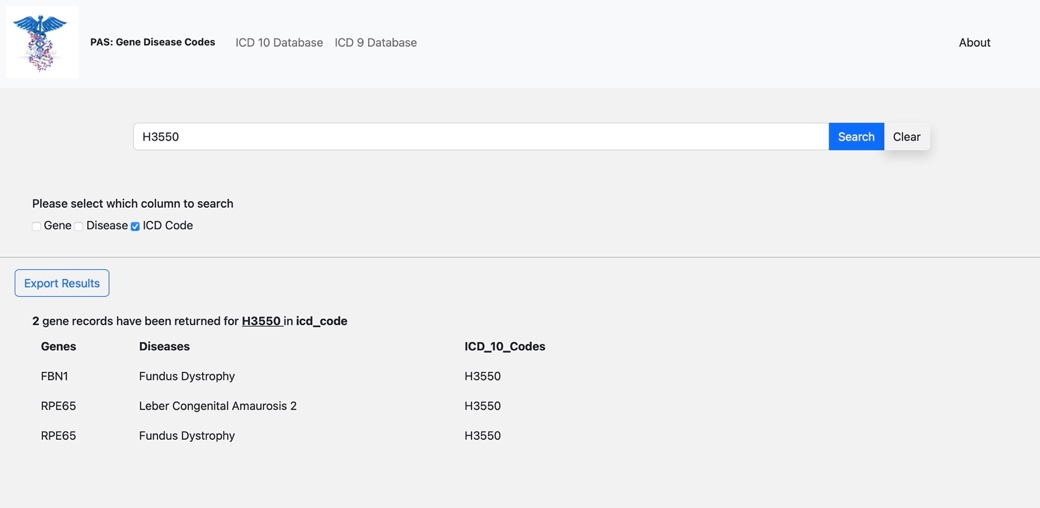


**S.** Figure 3. Results of searching ICD 10 Code (H3550)

When the user searches a valid ICD 10 code the results will be displayed. As per the search in S. Figure 3, the webpage relays all the genes and diseases associated with the ICD 10 code (H3550) and 2 gene records have been returned for the ICD 10 code (S. Figure 2 and 3).

### **ICD 9**


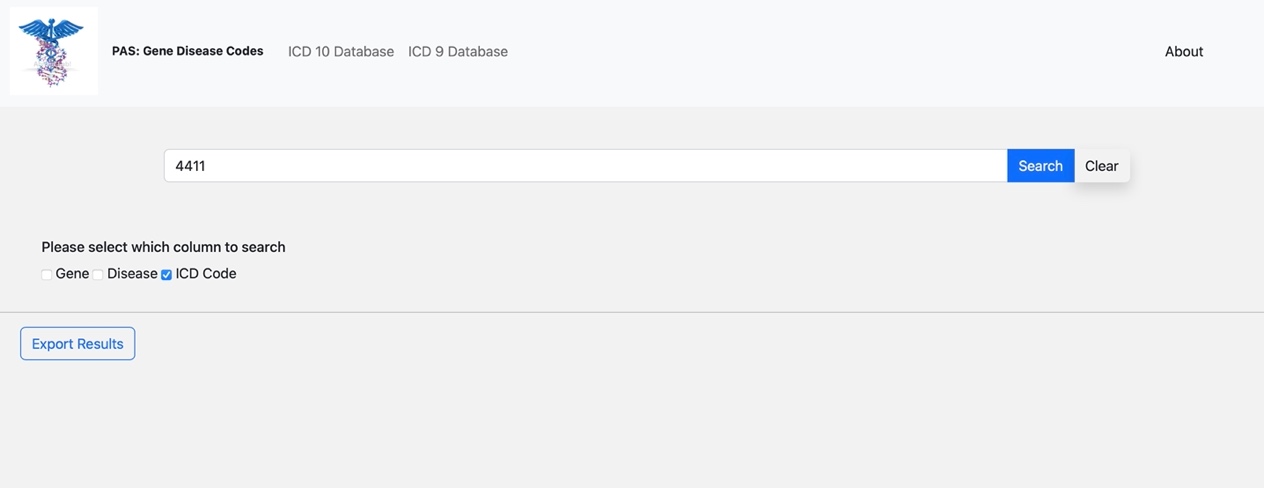


**S.** Figure 4. Searching of ICD 9 Code (4411)

When the user would like to search an ICD 9 code, the ICD 10 database will automatically be disabled when the ICD 9 text button is selected. The webpage will require the input of the ICD 9 code in the search bar by the user. The information will be relayed to our database and provide results based on the options chosen and the ICD code input by the user (S. Figure 4).


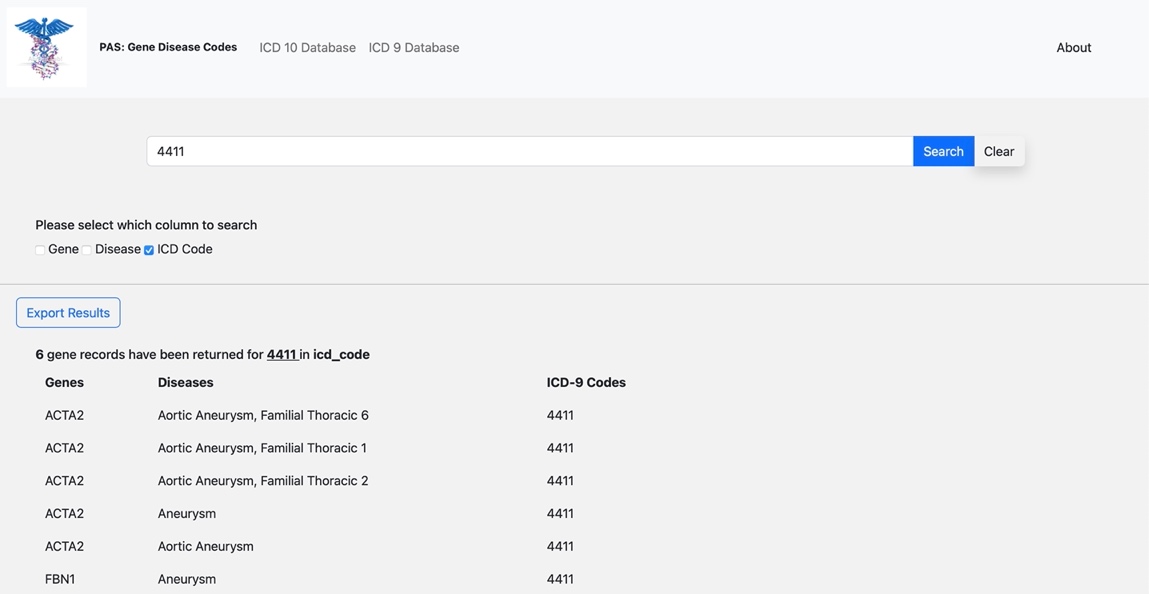


**S.** Figure 5. Results of searching ICD 9 Code (4411)

When the user searches a valid ICD 9 code the results will be displayed. As per the search in S. Figure 5, the webpage relays all the genes and diseases associated with the ICD 9 code (4411) and 6 gene records have been returned for the ICD 9 code.

## **Searching Using Gene Checkbox**


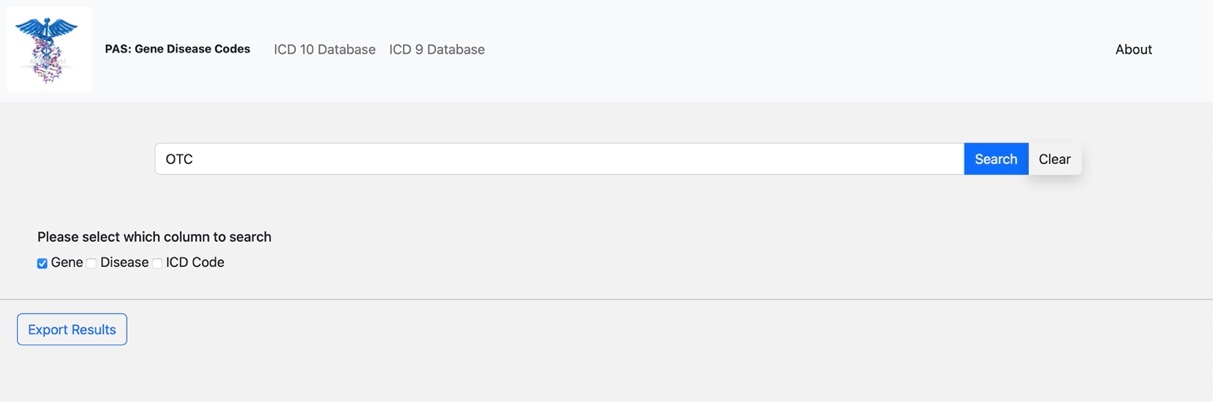


**S.** Figure 6. Searching of Gene (OTC)

The Webpage allows the user to search any of the 73 ACMG genes [1] and will provide the user results with the corresponding disease and ICD code associated with the gene. The user can also view the ICD 9 code (S. Figure 6) as well by clicking on the text button and ICD 10 database will be automatically disabled and other options will get unchecked (Disease and ICD code).


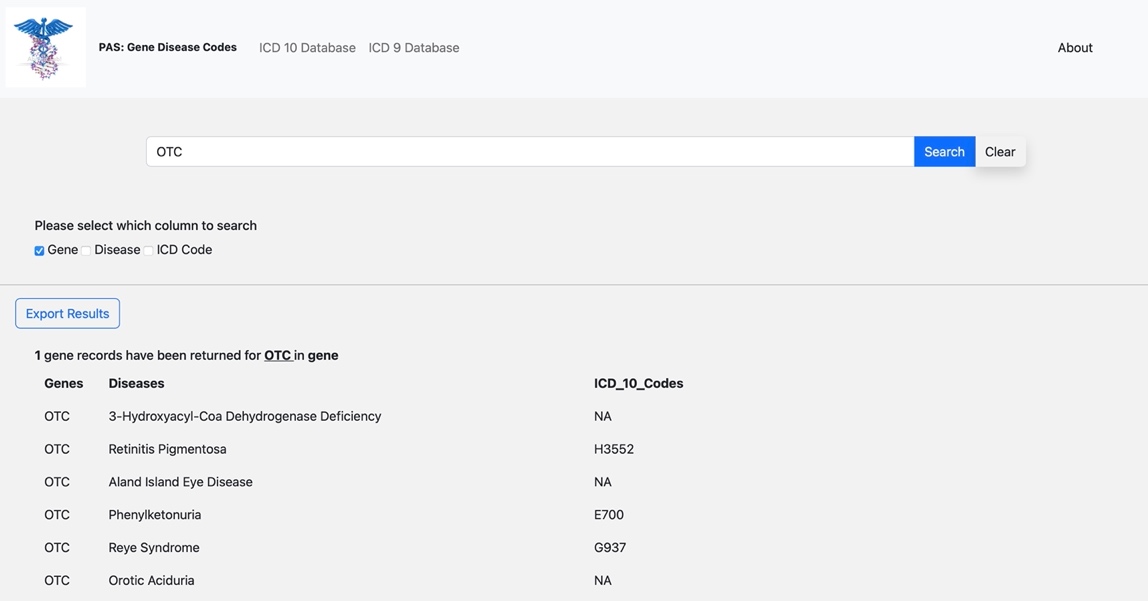


**S.** Figure 7. Results of searching Gene (OTC)

The webpage returns the result to the user specifying that there is 1 gene matching to the search input and provides all the diseases associated with the gene as well as the corresponding ICD codes. In S. Figure 7, the ICD code chosen is “ICD 10”, but the user can retrieve the corresponding ICD 9 code as well that is associated with the gene and disease (S. Figure 7).

## **Searching Using Disease Checkbox**


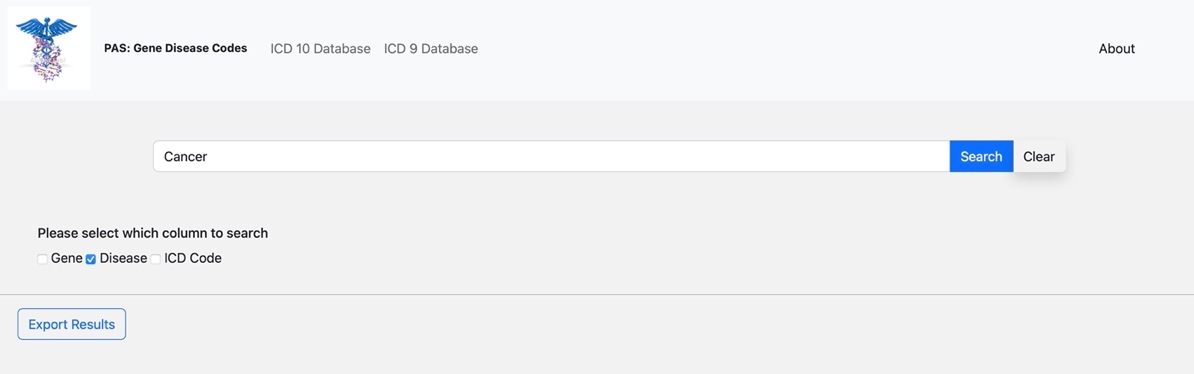


**S.** Figure 8. Searching of Disease (Cancer)

The webpage also allows the user to search for any diseases that are associated with the genes curated by reliable sources. The user has to key in the desired disease in the search bar and then click the “Disease” check box. Any other option (Gene or ICD code) will get unchecked, and the user can either click the search button or press ‘Enter/Return’ on their keyboard to view their results (S. Figure 8).


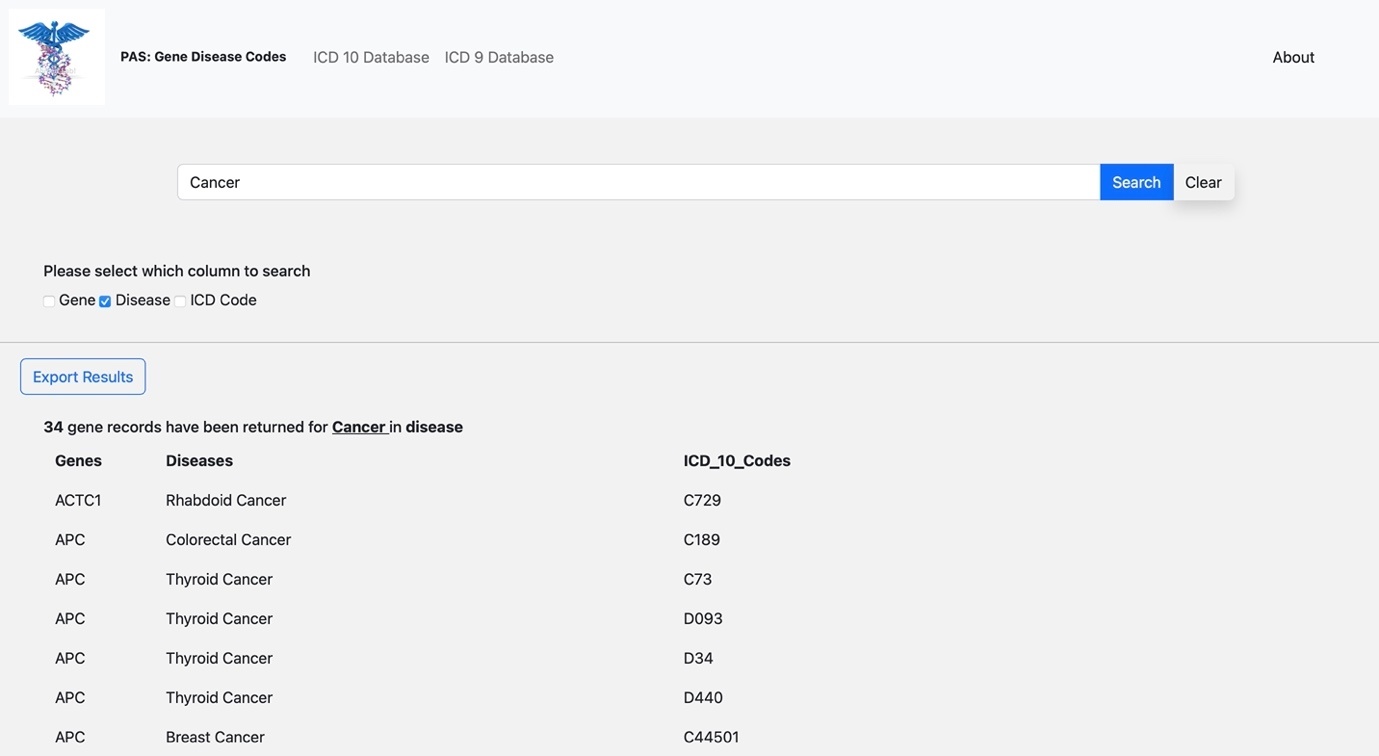


**S.** Figure 9. Results of Disease (Cancer)

As shown in S. Figure 9, the associated ICD Codes and Genes are displayed for the disease input, for which the webpage also shows that there are 34 genes associated with the keyword of the disease.

## **Exporting Results**


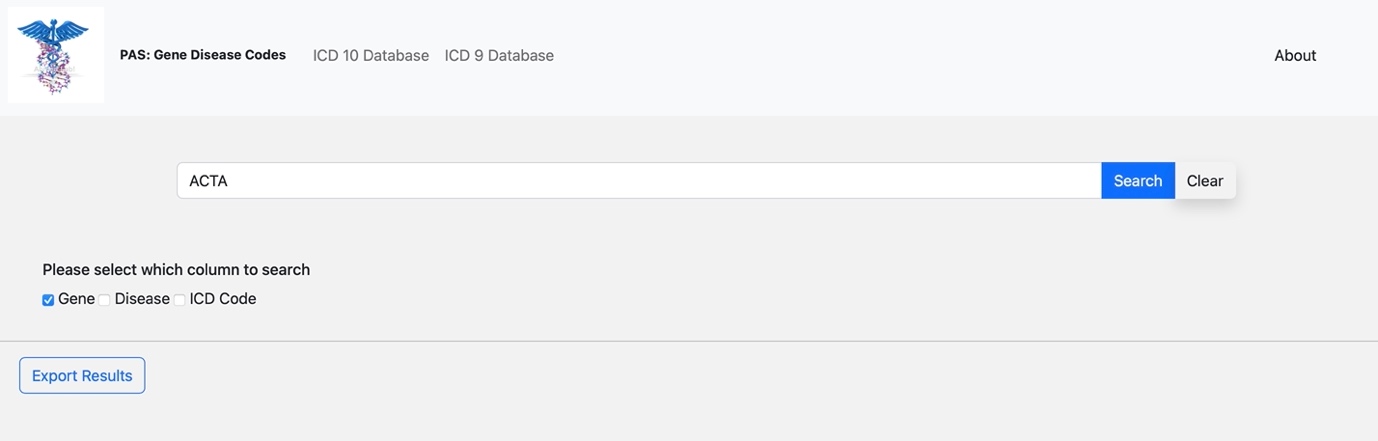


**S.** Figure 10. Searching of Keyword to Export Results

The webpage offers the functionality to “Export Results” as shown in S.Figure 10 and allow the users to key in their desired feature to search. In S.Figure 10, the Gene (ACTA) will be searched against the desired database, in the case of S.Figure 10, it will be searched in the ICD 10 database. The user has the option to search their keyword against the ICD 9 and Disease databases as well to export the results (S. Figure 10).


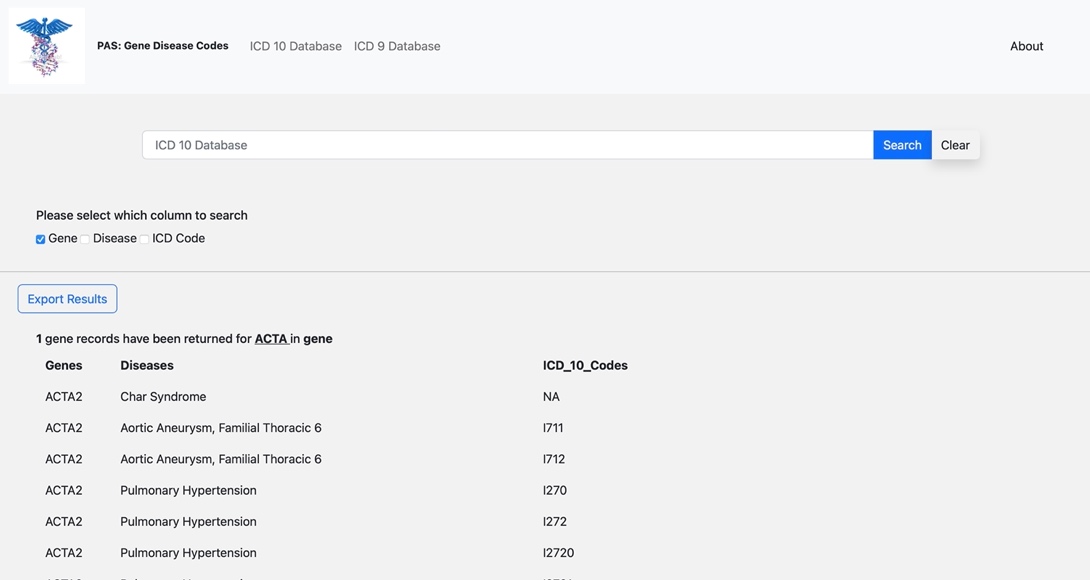


**S.** Figure 11. Preparing Webpage to Export Results

The webpage has retrieved results of the gene ACTA along with its associated diseases and ICD 10 codes as shown in S.Figure 11. The user can then click the “Export Results” button which will download a CSV (.csv) file locally on their device. The user can also view it using Microsoft Excel or Text Editor as they prefer (S. Figure 11).

## **Gene Disease Database: Graphical User Interface**

The website’s main functionality and focus is a user-friendly design focusing on ease of accessibility and functionality, ultimately allowing users to easily retrieve their desired output. Once the user has opened the webpage, the user simply has to select the option (ICD 9 or ICD 10) and checkbox according to the query they would like to search.


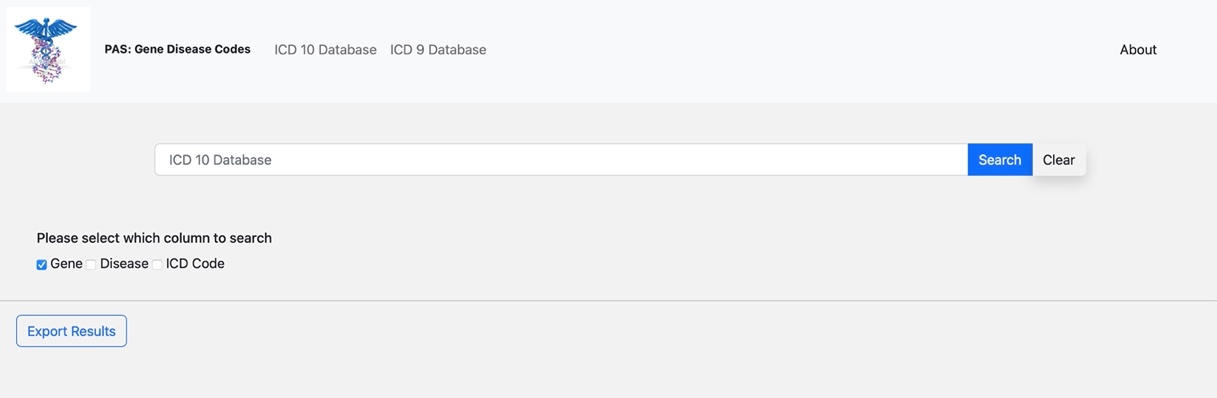


**S.** Figure 12. Graphical User Interface of Gene Disease Database

The user may click the search button to view their results or click the clear button to reset the webpage in their original state. If the user wishes to know more about the team behind the webpage, they can click the “About” page to view the team and learn more about Ahmed Lab (S. Figure 12).

## **Software Development**

The website’s source code is in PHP and HTML with CSS/Bootstrap to provide an aesthetically pleasing design for the user’s viewing. The webpage utilises a database in MYSQL; therefore, PHP is used as the server-side language to display the data in an organised manner to the user. In the future if there are any additions or removal to the ACMG Gene List [1], the developers have an easy and efficient method to update and provide current information to the users. CSS and Bootstrap provide a wide range of abilities which allow developers to improve and design the website to make it appealing to the users.

## **Gene Disease Database**

Gene Disease Database utilises the process of result retrieval through an SQL database, where the keyword is parsed in the backend to appropriately query and provide the result to the user. The Gene, Disease, and ICD codes were initially in an excel file, and we cleaned the file using a python script using Python programming language. The values were then extracted using pandas (library in Python), and the python script pairs each gene with a disease and ICD Code. These values are extracted to a text file and then put into a SQL procedure that produces all relationships.

|  | Genes | The Genes relationship contains the names of all 73[1] genes assigned to a unique ID. |
| --- | --- | --- |
|  | Diseases | The Diseases relationship contains the names of all diseases assigned to a unique ID. |
|  | ICD 10 | The ICD 10 relationship contains the names of all diseases assigned to a unique ID. |
|  | ICD 9 | The ICD 9 relationship contains the names of all diseases assigned to a unique ID. |
|  | Gene Disease | The Gene Disease relationship contains pairings of genes with diseases according to their mappings in the Excel File. |
|  | Gene Disease ICD 10 | The Gene Disease ICD 10 relationship contains pairings of genes, diseases, and ICD 10 codes according to their mappings in the excel file. This is the table being queried when the ICD 10 text button is selected. |
|  | Gene Disease ICD 9 | The Gene Disease ICD 9 relationship contains pairings of genes, diseases, and ICD 9 codes according to their mappings in the excel file. This is the table being queried when the ICD 9 text b is selected. |

**S. Table 2.** Relations

# **References**

1. Miller, D. T., Lee, K., Chung, W. K., Gordon, A. S., Herman, G. E., Klein, T. E., Stewart, D. R., Amendola, L. M., Adelman, K., Bale, S. J., Gollob, M. H., Harrison, S. M., Hershberger, R. E., McKelvey, K., Richards, C. S., Vlangos, C. N., Watson, M. S., Martin, C. L., & ACMG Secondary Findings Working Group (2021). ACMG SF v3.0 list for reporting of secondary findings in clinical exome and genome sequencing: a policy statement of the American College of Medical Genetics and Genomics (ACMG). *Genetics in medicine : official journal of the American College of Medical Genetics*, *23*(8), 1381–1390.
